# Serum lipid alterations in oral squamous cell carcinoma and oral potentially malignant disorders: A systematic review and meta-analysis

**DOI:** 10.1038/s41598-026-46163-z

**Published:** 2026-03-31

**Authors:** Shreeja Kishor, Mila Janjic Rankovic, Sven Otto, Tamara Katharina Kakoschke

**Affiliations:** 1 Department of Oral and Maxillofacial Surgery and Facial Plastic Surgery, University Hospital, LMU Munich, Lindwurmstrasse 2a, 80337 Munich, Germany; 2 Department of Orthodontics and Dentofacial Orthopedics, LMU University Hospital, LMU Munich, 80336 Munich, Germany

**Keywords:** Lipid profile, Oral squamous cell carcinoma (OSCC), Oral potentially malignant disorders (OPMD), Dyslipidemia, Cholesterol, High-density lipoprotein, Meta-analysis, Systematic review, Triglycerides, Biomarkers, Cancer, Diseases, Medical research, Oncology, Risk factors

## Abstract

Alterations in serum lipids have been linked to systemic and neoplastic diseases, including oral malignancies. Lipid disturbances are reported inconsistently in oral potentially malignant disorders (OPMD) and oral squamous cell carcinoma (OSCC). This systematic review and meta-analysis clarifies lipid profile patterns across OPMD, OSCC, and healthy controls. Following PRISMA 2020 guidelines and PROSPERO registration, a comprehensive database search through November 2025 identified fifty-two eligible case-control studies comparing total cholesterol (TC), high-density lipoprotein (HDL), low-density lipoprotein (LDL), very-low-density lipoprotein (VLDL), and triglycerides (TGs) between OPMD, OSCC, and healthy individuals. Data were synthesized using a random-effects model, Cochran’s Q and I² statistics for heterogeneity and Risk of Bias. This meta-analysis showed significantly reduced TC, HDL, LDL, VLDL, and TGs in both OSCC and OPMD compared with healthy controls (*p* < 0.001), with greater reductions in OSCC. Direct comparisons between OSCC and OPMD revealed significant differences in TC (*p* = 0.025) and LDL (*p* = 0.04), while HDL and VLDL differences were not significant. High heterogeneity and mild publication bias for TC and TGs were observed. Results indicate an association between lipid profiles and disease stage, but prospective studies are needed to determine causality and diagnostic utility.

**Supplementary Information:**

The online version contains supplementary material available at 10.1038/s41598-026-46163-z.

## Introduction

Serum lipids are fundamental biomolecules that reflect systemic lipid metabolism and play essential roles in energy transport, storage, cellular signaling^1–3^. Alterations in circulating lipid levels reflect broader changes in lipid metabolism and have been increasingly implicated in a variety of disease processes^4,5^. Dyslipidemia, for example, abnormal levels of triglycerides (TGs), low-density lipoproteins (LDL), high-density lipoproteins (HDL), and other lipoprotein fractions are well-established risk factors for cardiovascular disorders such as atherosclerosis, myocardial infarction, and stroke^6^.

Similarly, lipid dysregulation contributes to the pathogenesis of metabolic syndromes, including diabetes mellitus, where it promotes insulin resistance and accelerates disease progression^7^. In non-alcoholic fatty liver disease, excessive lipid accumulation leads to hepatocellular damage and chronic inflammation^8^. Accumulating data implicate serum lipid dysregulation in the metabolic reprogramming of cancer. Tumor cells frequently rewire their lipid metabolism to satisfy increased demands for membrane synthesis, rapid energy generation, and signalling, and these changes can be mirrored by altered serum lipid profiles^9,10^.

A few studies have documented decreased levels of total cholesterol (TC), HDL, and LDL in patients with oral squamous cell carcinoma (OSCC) compared to healthy controls^11–13^. Mass spectrometry-based lipidomic analyses of OSCC tissue further reveal substantial shifts in glycerophospholipid and cholesterol metabolism, suggesting that cancer-associated metabolic reprogramming extends deeply into lipid homeostasis^14^. Serum lipid alterations have been documented in head and neck cancer patients during disease progression and treatment, including chemotherapy and radiotherapy^15,16^.

Moreover, in oral potentially malignant disorders (OPMD) such as leukoplakia (LK), erythroplakia (EP), lichen planus (LP), or oral submucous fibrosis (OSMF), altered serum lipids have also been reported^17–22^. These findings raise the possibility that lipid alternations begin early in the neoplastic process, perhaps as a response to increased lipid utilization by proliferating dysplastic cells. Indeed, some authors propose that hypolipidemia in premalignant and malignant oral lesions may reflect the consumption of lipids for new membrane biosynthesis^22–24^.

Some studies report consistent lipid reductions, while others note more complete changes, including shifts in specific lipoprotein subclasses^22,25,26^. Moreover, many investigations do not stratify clearly between pre-malignant and malignant stages, which may obscure disease-stage-specific patterns. These discrepancies highlight the need for a comprehensive analysis to clarify the role of lipid alterations in oral carcinogenesis. Given the distinct pathophysiological mechanisms of OPMD and OSCC, meaningful interpretation requires stratified analyses rather than pooling pre-malignant and malignant conditions together. The present review aim to address this gap.

This systematic review and meta-analysis synthesizes current evidence on alterations in serum lipid profiles (TC, HDL, LDL, VLDL, and TGs) in patients with OPMD and OSCC compared with healthy controls. In addition, subgroup analyses are performed to examine differences between pre-malignant and malignant conditions and to assess whether lipid alterations follow a continuum or represent condition-specific patterns.

## Methods

This systematic review process adheres to the PRISMA 2020 (Preferred Reporting Items for Systematic Reviews and Meta-Analyses) guidelines^27,28^, to ensure a thorough and systematic assessment of the literature, and was previously registered on PROSPERO (ID: CRD420251048818).

### Eligibility criteria

Inclusion criteria were defined in accordance with the PICO framework. Population (P) included patients diagnosed with the oral malignant conditions of OSCC and OPMD, LK, EP, OSMF, LP, along with matched healthy controls. The Intervention (I) involved quantitative assessment of serum lipid parameters, including TC, LDL, HDL, VLDL, and TGs, determined by validated biochemical methods. Comparison (C) was made with healthy control groups without oral cancer, precancerous lesions, or other head and neck malignancies. Outcome (O) focused on alterations in lipid profiles, in relation to OPMD and oral malignancies.

Studies were included if they: (1) evaluated the association between lipid profiles (TC, LDL, HDL, VLDL, TGs) and oral malignant or pre-malignant conditions, (2) involved both patient groups (oral cancer and/or potentially malignant oral lesions) and healthy controls, (3) reported quantitative lipid measurements using standardized biochemical methods, (4) included at least 30 participants, (5) provided extractable quantitative data suitable for meta-analysis, and (6) were peer-reviewed articles published in English. Studies were excluded if they: (1) did not assess lipid profiles in relation to oral malignant or pre-malignant conditions, (2) enrolled fewer than 30 participants, (3) focused on unrelated or broader aspects of lipid metabolism, (4) used animal models or in vitro designs, (5) reported lipid data in non-standardized or incompatible formats, (6) were reviews, editorials, case reports, or other ineligible study designs, (7) were available only as conference abstracts without adequate methodological or quantitative details, or (8) did not provide extractable or analysable data.

### Search strategy and study selection process

A literature search was conducted in PubMed, Embase, and Scopus databases to identify eligible studies evaluating lipid profiles in relation to oral malignant and pre malignant conditions. The search included articles published up to Nov 2025. Based on the research question and the predefined eligibility criteria, a comprehensive search strategy was developed. In alignment with Cochrane recommendations for studies focused on clinical topics the search approach was structured around a core concept, which was then divided into three sub-concepts for precise and effective search terms^29^ (Table 1).

**Table 1:** Concept of the search strategy.

Domain	Search term
Field (Exposure)	“lipid profile” OR “serum lipids” OR “lipids” OR “lipid analysis” OR cholesterol OR triglycerides OR lipoproteins OR HDL OR LDL OR VLDL
	AND
Condition (Population)	“oral malignancy” OR “oral cancer” OR “oral squamous cell carcinoma” OR “oral premalignant lesions” OR leukoplakia OR erythroplakia OR “oral submucous fibrosis” OR “lichen planus”
	AND
Sample/Method	“blood sample” OR “serum” OR “plasma” OR “serum lipids”

Search strategies were customized for each database using appropriate syntax, controlled vocabulary, and indexing systems. This structured approach was implemented across three major sources PubMed, Embase, and Scopus with specific adaptations applied to suit the requirements and functionalities of each database (Table 2). Boolean operators “AND” and “OR” were consistently applied across databases. In addition to the database searches, the reference lists of all included articles and relevant reviews were manually screened to identify any additional studies meeting the inclusion criteria.

**Table 2:** Overview of search strategies across databases and the corresponding number of records retrieved.

Database	Search strategy	Results
PubMed	((“lipid profile” OR “serum lipids” OR “lipids” OR “lipid analysis” OR “cholesterol” OR “triglycerides” OR “lipoproteins” OR “HDL” OR “LDL” OR “VLDL”) AND (“oral cancer” OR “oral squamous cell carcinoma” OR “head and neck cancer” OR “oral potentially malignant disorders” OR “oral premalignant lesions” OR “leukoplakia” OR “erythroplakia” OR “oral submucous fibrosis” OR “lichen planus”))	334
Embase	(‘lipid profile’/exp OR ‘lipids’/exp OR ‘serum lipids’/exp OR ‘cholesterol’/exp OR ‘triglyceride’/exp OR ‘lipoprotein’/exp OR ‘high density lipoprotein’/exp OR ‘low density lipoprotein’/exp OR ‘very low density lipoprotein’/exp) AND (‘oral cancer’/exp OR ‘oral squamous cell carcinoma’/exp OR ‘head and neck cancer’/exp OR ‘oral premalignant lesion’/exp OR ‘leukoplakia’/exp OR ‘erythroplakia’/exp OR ‘oral submucous fibrosis’/exp OR ‘lichen planus’/exp)	258
Scopus	TITLE-ABS-KEY (“lipid profile” OR “serum lipids” OR “lipids” OR “cholesterol” OR “triglycerides” OR “HDL” OR “LDL” OR “VLDL”) AND (“oral cancer” OR “oral squamous cell carcinoma” OR “head and neck cancer” OR “oral potentially malignant disorders” OR “oral premalignant lesions” OR leukoplakia OR erythroplakia OR “oral submucous fibrosis” OR “lichen planus”)	180

To ensure completeness of the search strategy, we additionally verified the PubMed search using controlled vocabulary from the Medical Subject Headings (MeSH) database, including terms such as “Blood Lipids,” “Mouth Neoplasms,” and “Precancerous conditions.”This verification search did not identify any additional eligible studies beyond those already captured by the original search strategy. All identified citations were imported into EndNote 21 (Clarivate Analytics, Philadelphia, PA, USA), and duplicates were automatically removed using the software’s built-in deduplication tool, followed by a manual verification. The initial screening phase involved independent evaluation of titles and abstracts by two reviewers (S.K., T.K.K.) to assess alignment with the predefined inclusion criteria. Disagreements during the selection process were resolved by discussion and consensus. In the second phase, the full texts of the selected references were retrieved and evaluated in detail. Studies that failed to meet the inclusion criteria or lacked usable data were excluded.

### Data extraction

Data from the included studies were independently extracted by both reviewers using specially designed data extraction sheets. Any discrepancies between the extracted data were resolved through discussion until consensus was achieved. For each study that met the final eligibility criteria, the following data were extracted: first author’s name, publication year, disease type, number of participants per group, age and sex distribution, sample type and collection method; lipid quantification techniques used, serum levels of TC, HDL, LDL, VLDL and TGs measured in mg/dl and reported as mean ± standard deviation (SD). All extracted data were organized into Excel spreadsheets (Excel 2010, Microsoft Corporation, Redmond, WA, USA). Data entry was independently checked twice by both reviewers prior to further analysis (S.K., T.K.K.). Any discrepancies were resolved through discussion, or by a third reviewer (S.O.).

### Risk of bias (RoB) assessment in included studies

The studies included in this review were observational case control in design, but the primary methodological concerns were related to measurement and outcome ascertainment processes. A domain-based, adapted RoB assessment was developed, drawing on principles from established frameworks (such as QUADAS2), to evaluate bias sources relevant to observational case control studies. The assessment included the following domains: bias due to selection of participants in the study, bias due to limited sample size, blinding-related bias, bias arising from measurement of exposure and outcome, bias due to confounding, and bias in the selection of reported results. Each study was then assigned an overall RoB (low, moderate, or serious) based on the cumulative assessment across domains. This approach allows for a more detailed, domain-based analysis of individual sources of bias and it clearly identifies where the weaknesses of each study lie and avoids the aggregated “score” approach of Newcastle–Ottawa Scale, which may hide specific methodological limitations. In addition, the generic RoB tool^30^ was adapted for visualization and generation of the RoB figure. The RoB assessments were conducted independently by two authors (S.K. and T.K.K.) and reviewed with third author (S.O.).

### Meta-analysis and synthesis of results

Mean values and their respective SD of serum lipid levels were extracted for each lipid fraction of interest. For studies reporting multiple mean values per lipid fraction, separate entries were created for inclusion in the analyses to ensure accurate representation of the data. Wherever necessary, the control sample size was divided proportionally across comparisons in accordance with Cochrane Handbook^29^ to avoid double-counting participants. Heterogeneity among studies was evaluated using Cochran’s Q-statistic, which assesses whether outcome variations were due to chance, and the I²-statistic, which quantifies the proportion of variation attributable to heterogeneity rather than sampling error. A random-effects model was used to account for variability across studies, with the standardized mean difference (SMD) serving as the effect size, and a p-value of less than 0.05 was considered statistically significant. Robustness was assessed using a leave-one-out sensitivity analysis. The forest plot visually summarized the meta-analysis, with the diamond representing the pooled effect size and 95% confidence interval (CI), allowing for clear visualization of aggregated findings across studies. Funnel plot were generated to assess potential publication bias in the included studies. Additionally, meta-regression examined study-level moderators influencing variability in effect sizes across included studies. All statistical analyses and visualizations were conducted using R Studio version (2024.09.1 + 394).

## Results

### Literature search

**Fig. 1:**
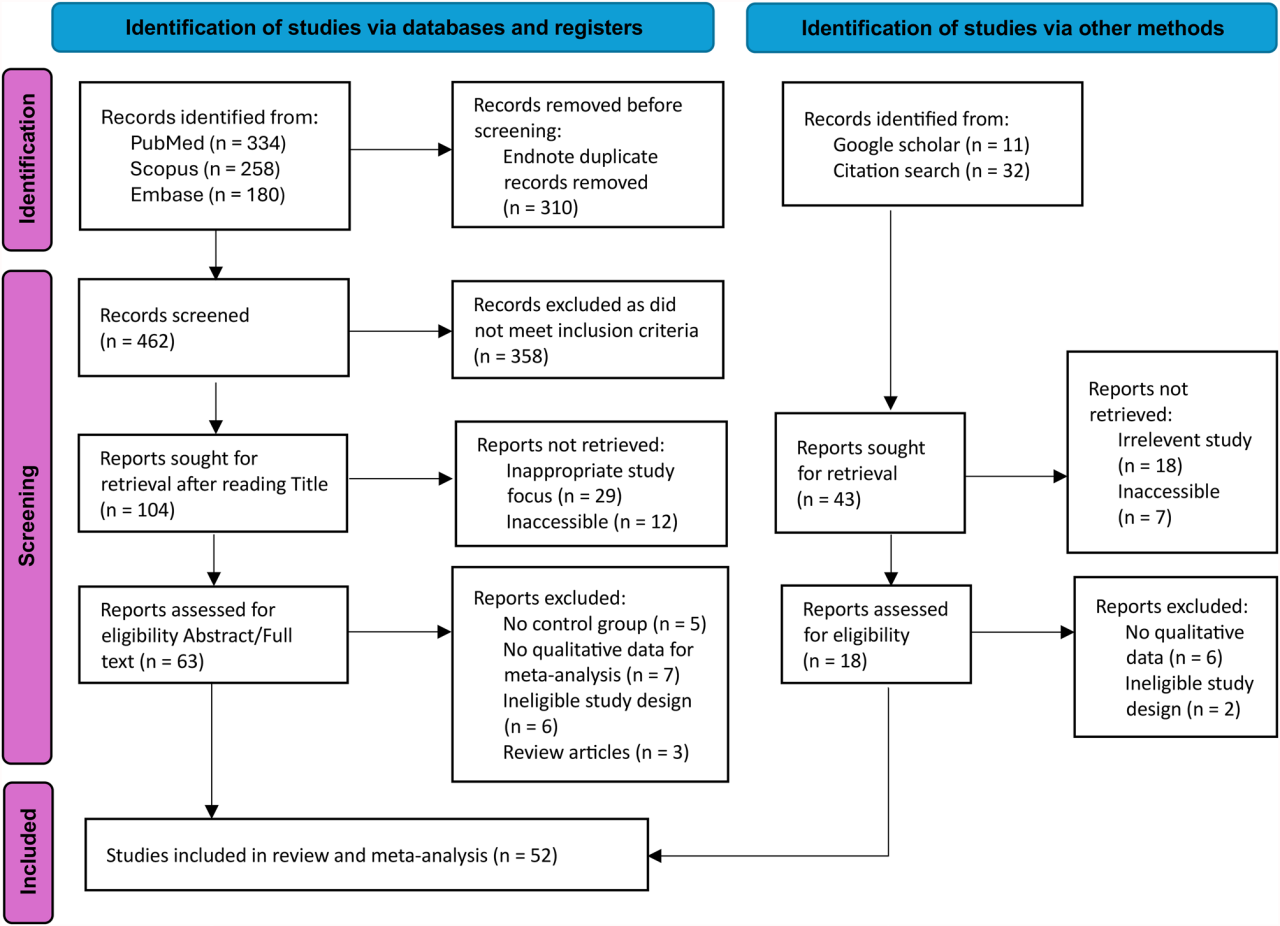
The Literature search Preferred reporting items for systematic reviews and meta-analyses (PRISMA) flow diagram.

The study selection process is summarized in the PRISMA 2020 flow diagram (Fig. 1). A total of 772 records were identified across three sources: PubMed (*n* = 334), Scopus (*n* = 258), and Embase (*n* = 180). Additional 43 studies were identified through Google scholar and citation search.

The records identified and removed in each stage are documented in the PRISMA flow chart in Fig. 1. As could be seen, the title and abstracts of 147 records (104 + 43) were manually screened for exclusion criteria. Preliminary evaluation of abstracts suggested that several of these studies (29 + 18 = 47) focused on lipid alterations in other head and neck diseases rather than the serum lipid parameters relevant to the present review. Remaining 19 articles could not be accessed through the available databases and institutional subscriptions despite attempts to obtain the full texts through alternative sources, including Google scholar, Research gate, and contacting the corresponding authors. Consequently, full texts of 81 records were retrieved and assessed for eligibility. Of these, 29 were excluded for the following reasons: absence of a control group (*n* = 5)^31–35^, lack of quantitative data suitable for meta-analysis (*n* = 13)^18,19,36–46^, ineligible study design (*n* = 8)^47–54^, and review articles (*n* = 3)^55–57^(Supplementary Table S1). Finally, fifty-two studies were qualitataively reviewed and provided sufficient data for quantitative meta-analysis (Supplementary Table S2).

## Results of the RoB assessment

**Fig. 2:**
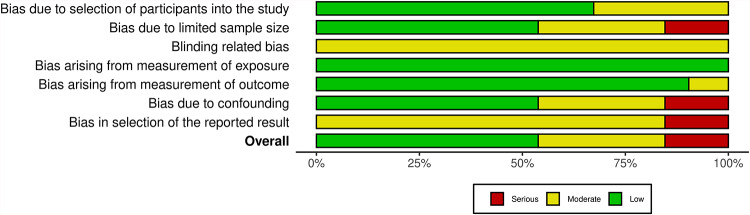
Risk of Bias assessment for all the included studies.

Out of all included studies, 28 papers demonstrated a low overall RoB^11–13,17,22–25,58–77^, primarily because they used standardized biochemical methods, matched controls, and objective outcome measures (Fig. 2). Sixteen studies were judged to have a moderate RoB^26,78–92^, often due to small, single-centre samples, convenience sampling, and incomplete adjustment for confounding factors such as tobacco use or diet. Eight studies exhibited a serious RoB^21,93–99^, mainly related to very small or unbalanced samples, non-random recruitment, and absence of blinding. There were minor concerns about confounding bias and limited sample size. Most of the studies had minimized outcome and verification bias through objective laboratory analyses but suffered from moderate to serious result selection bias and blinding bias. Consequently, while internal validity was generally fair, external validity and causal inference were limited across the evidence base (Supplementary Table S3).

### Study characteristics and results of individual studies

Fifty-two studies were included in the qualitative review, all of which were case control in design. Fourty seven studies were conducted in various regions of India, 2 studies were conducted among China population and one study each evaluated the Pakistan, Syria and Iran population. All the studies reported lipid profiles in OSCC, pre-malignant lesions, and healthy controls. The detailed characteristics of eligible studies structured using the PICO framework^27^ are presented in Supplementary Table S2.

### Heterogeneity

A total of 52 studies providing specific and adequate data for OSCC, OPMD, and corresponding control groups were included in the quantitative analysis for heterogeneity. The Cochrane’s Q-test indicated significant heterogeneity in true outcomes for TC, HDL, LDL, VLDL, and TGs (Table 3).

**Table 3:** Heterogeneity statistics of oral squamous cell carcinoma; OSCC with healthy control and oral potentially malignant disorder; OPMD.

For comparison between OSCC patients and healthy controls							
Parameters	Tau^2^	I^2^	H^2^	Df	Q	*p*	95% prediction interval for the true outcome
TC	3.32 (SE = 0.81)	98.19%	55.37	36	1106.90	< 0.0001	[-2.1740 to -0.9230]
HDL	7.33 (SE = 1.76)	99.13%	114.37	36	1437.86	< 0.0001	[-2.8896 to -1.0406]
LDL	2.51 (SE = 0.64)	98.07%	51.88	33	382.86	< 0.0001	[-1.6042 to -0.3218]
VLDL	3.98 (SE = 1.09)	98.45%	64.59	28	659.12	< 0.0001	[-1.6380 to -0.0103]
TGs	1.80 (SE = 0.46)	97.13%	34.89	34	731.13	< 0.0001	[-1.3039 to -0.3492]
For comparison between OPMD patients and healthy controls							
Parameters	Tau^2^	I^2^	H^2^	Df	Q	p	95% prediction interval for the true outcome
TC	2.01 (SE = 0.54)	96.53%	28.81	30	671.60	< 0.0001	[-1.9055 to -0.8288]
HDL	3.41 (SE = 0.91)	97.84%	46.31	30	920.70	< 0.0001	[-2.1576 to -0.7735]
LDL	1.80 (SE = 0.51)	96.65%	29.84	27	504.63	< 0.0001	[-1.2858 to -0.2110]
VLDL	2.07 (SE = 0.60)	97.03%	33.65	26	516.65	< 0.0001	[-1.1917 to -0.0180]
TGs	1.43 (SE = 0.40)	95.77%	23.64	28	509.47	< 0.0001	[-1.1658 to -0.2221]
For comparison between OSCC patients and OPMD							
Parameters	Tau^2^	I^2^	H^2^	Df	Q	p	95% prediction interval for the true outcome
TC	1.12 (SE = 0.44)	95.55%	22.46	15	185.04	< 0.0001	[-1.2106 to -0.0047]
HDL	2.23 (SE = 0.85)	97.63%	42.20	15	310.49	< 0.0001	[-1.0234 to 0.6203]
LDL	3.53 (SE = 1.49)	98.48%	65.93	12	250.00	< 0.0001	[-2.2538 to 0.1090]
VLDL	1.66 (SE = 0.74)	96.88%	32.05	11	284.07	< 0.0001	[-1.4684 to 0.2254]
TGs	2.40 (SE = 0.94)	97.58%	41.33	14	389.14	< 0.0001	[-1.6103 to 0.1521]

### Serum Lipid Levels Between OSCC and Healthy Controls

**Fig. 3:**
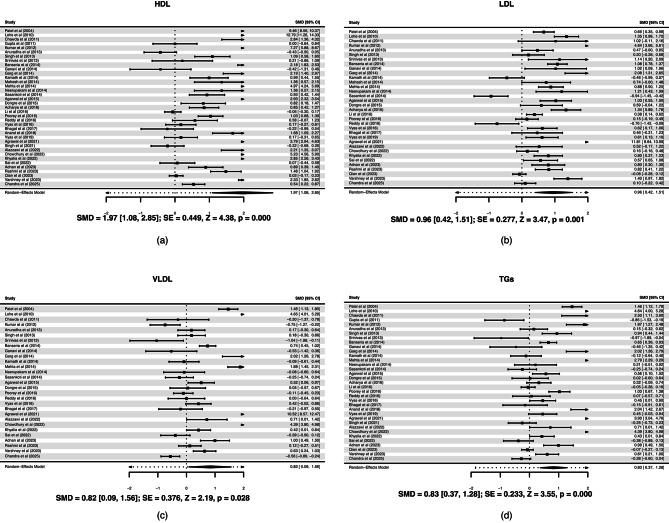
Forest plot between oral squamous cell carcinoma; OSCC and healthy controls for (**a**) High-density lipoproteins; HDL, (**b**) Low-density lipoproteins; LDL, (**c**) Very low-density lipoproteins; VLDL, and (**d**) Triglycerides; TGs.

A total of 37 studies compared the lipids profile of OSCC (*n* = 1855) and healthy controls (*n* = 1749). Cancer patients showed lower HDL, LDL, VLDL, and TGs compared to healthy controls and the difference in each case was statistically significant. The forest plots are shown in Fig. 3. The spread of study estimates (including positives and very large negatives) suggests substantial heterogeneity.

TC level is shown in Fig. 5(a). The positive SMD (1.55) suggests that TC is significantly lower in OSCC patients compared to healthy controls. Since the comparison is “Cancer − Control,” a positive SMD indicates that the control group has higher TC. The 95% CI does not cross zero, and the p-value < 0.001, confirming a strong, statistically significant difference. This difference is large (SMD > 0.8). The plot shows wide variation among studies, with some extreme SMDs (e.g., > 6 in a few studies^58,93^, indicating substantial heterogeneity. The asymmetry in funnel plot implies some degree of bias, but it could also reflect true heterogeneity due to differences in population, assay methods, or cancer stage across studies (Fig. 5(b)). We conducted leave-one-out sensitivity analysis for each of the lipids and no significant change was noted (Supplementary Figures S1–S6).

### Serum lipid levels between OPMD and healthy controls

**Fig. 4:**
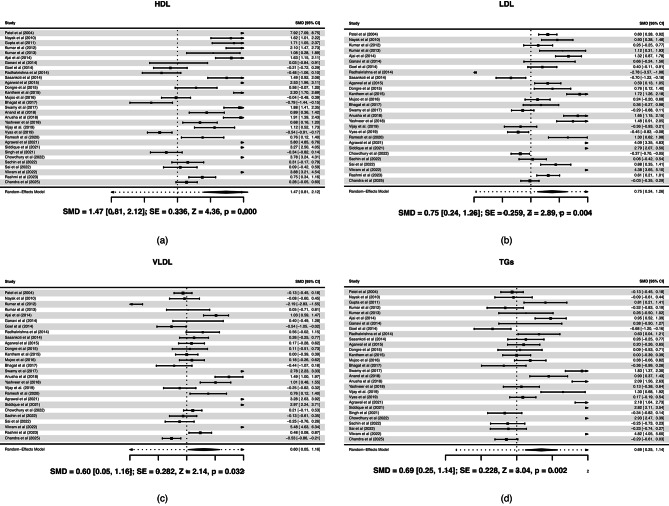
Forest plot between oral potentially malignant disorder; OPMD and healthy controls for (**a**) High-density lipoproteins; HDL, (**b**) Low-density lipoproteins; LDL, (**c**) Very low-density lipoproteins; VLDL, and (**d**) Triglycerides; TGs.

Statistically significant reduction in TC, HDL, LDL, VLDL, and TGs levels among patients with OPMD compared with healthy controls. A total of 31 studies were included, comprising 1398 patients with OPMD and 1129 healthy controls. The forest plots are shown in Fig. 4. The positive and significant SMD (*p* < 0.001, effect size = 1.47) with HDL levels indicates a large, statistically significant lower HDL in OPMD compared to healthy controls. LDL levels are also lower in OPMD compared to healthy controls. However, the effect size (0.75) is moderate, smaller than for HDL, indicating less pronounced but still meaningful reduction. For VLDL, the SMD is positive and significant (*p* = 0.032), again suggesting lower VLDL levels in OPMD compared to controls. The magnitude of change in VLDL is smaller than HDL or LDL changes. With TGs the SMD is positive and statistically significant (*p* = 0.002), showing that TGs are also statistically significantly lower in OPMD patients. The effect of TGs is moderate (SMD = 0.69), consistent with the LDL and VLDL results.

**Fig. 5:**
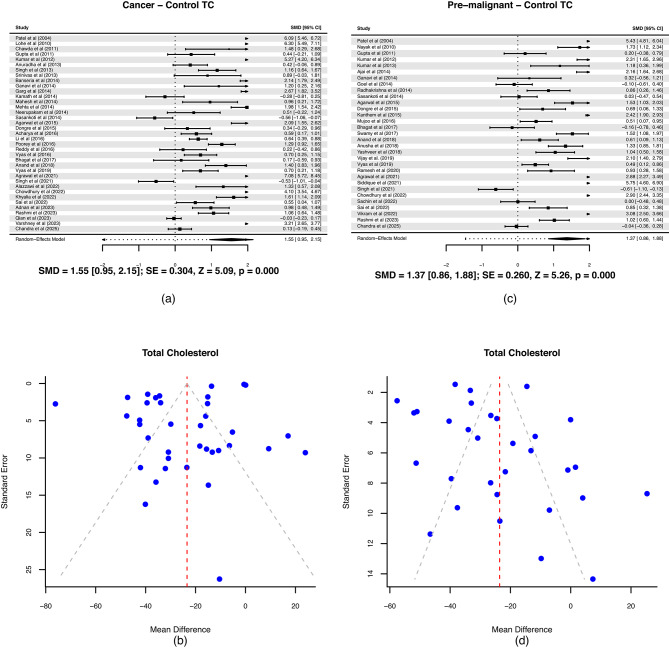
(**a**) Forest plot between oral squamous cell carcinoma; OSCC and healthy controls for Total cholesterol; TC, (**b**) Funnel plot between OSCC and healthy controls for TC, (**c**) Forest plot between oral potentially malignant disorder; OPMD and healthy controls for TC, (**d**) Funnel plot between OPMD and healthy controls for TC.

The positive SMD value in Fig. 5(c) indicates that TC levels are significantly lower in OPMD patients compared to healthy controls. The difference is large and highly significant (*p* < 0.001). The overlap in magnitude between this result (SMD 1.37) and the cancer vs. control result (SMD 1.55) suggests that cholesterol decline may already be present at the premalignant stage. Some studies^73,76^, reported extremely high effect sizes, indicating moderate to high heterogeneity across studies. The funnel plot in Fig. 5(d) shows slight asymmetry, which may reflect mild publication bias or variation among studies. However, overall distribution of study results around the central mean difference suggests that any bias is likely limited. These findings are consistent with the possibility that lipid metabolism alterations may already be present in premalignant stages, although causal or temporal relationships cannot be established from cross-sectional data.

### Serum lipid levels between OSCC and OPMD

**Fig. 6:**
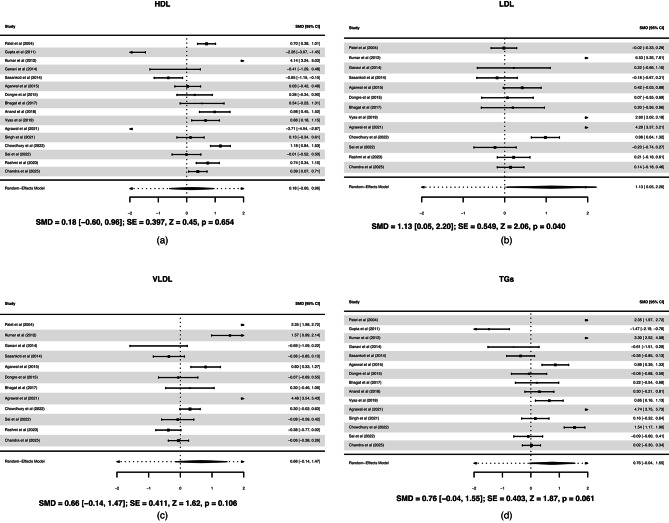
Forest plot between oral squamous cell carcinoma; OSCC and oral potentially malignant disorder; OPMD for (**a**) High-density lipoproteins; HDL, (**b**) Low-density lipoproteins; LDL, (**c**) Very low-density lipoproteins; VLDL, and (**d**) Triglycerides; TGs.

**Fig. 7:**
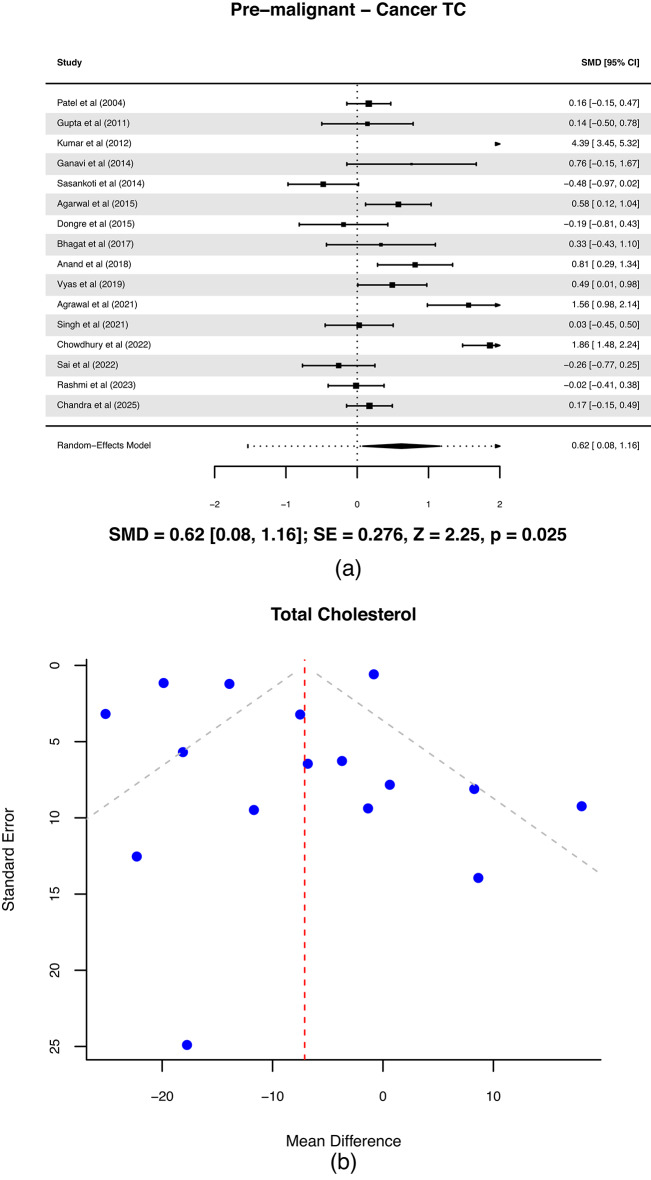
(**a**) Forest plot between oral squamous cell carcinoma; OSCC and oral potentially malignant disorder; OPMD for Total cholesterol; TC, (**b**) Funnel plot between OSCC and OPMD for TC.

Sixteen studies comparing lipid profiles of OSCC with OPMD, including 745 OPMD patients and 701 OSCC patients was analyzed. The resulting comparison of HDL, LDL, VLDL and TGs levels between these groups is shown in Fig. 6 with the TC difference in Fig. 7(a). A statistically significant difference was observed in TC (*p* = 0.025) and LDL (*p* = 0.04) levels, with TGs showing marginal significance (*p* = 0.06). In contrast, HDL (*p* = 0.65) and VLDL (*p* = 0.10) levels did not differ significantly between patients with OPMD and those with OSCC. LDL demonstrated a significant reduction in cancer patients compared to OPMD (SMD = 1.13; 95% CI: 0.05 to 2.20; *p* = 0.040), reflecting a moderate to large effect size, though the wide CI suggests influence from outlier studies.

In the TC levels plot (Fig. 7(a)), the positive SMD indicates that TC levels are significantly higher in OPMD patients than in those with OSCC, with a p-value of 0.025 confirming statistical significance. The CI does not cross zero, reinforcing that this difference is unlikely due to chance. Few studies^61,65,75^ reported strong positive effects, while a few^84,85^ showed minimal or negative differences, suggesting moderate heterogeneity and justifying the use of a random-effects model. The funnel plot (Fig. 7(b)) displays asymmetry, with smaller studies showing greater variation in effect sizes, indicating a mild publication bias or small study effect. Additionally, we conducted meta-regression analysis on studies with OPMD, OSCC and control groups which indicated that the meta-regression model provides no improvement over a standard random-effects meta-anlaysis model (Supplementary Figures S7–S11).

### Publication bias

Funnel plots were generated to assess potential publication bias across studies. In OSCC versus healthy controls, slight asymmetry was observed for HDL, suggesting potential small study effects, while LDL and TGs plots appeared symmetric, indicating low risk of publication bias. VLDL showed some asymmetry toward negative mean differences, which may reflect heterogeneity or selective reporting. For OPMD versus healthy controls, HDL and TGs funnel plots were largely symmetric, whereas LDL and VLDL showed minor asymmetry, suggesting possible small study effects. In the comparison between OPMD and OSCC, HDL exhibited notable asymmetry, indicating potential publication bias, whereas LDL, VLDL, and TGs were relatively balanced. The visual inspection suggests mild to moderate publication bias in certain comparisons, but the overall effect estimates are unlikely to be substantially affected (Supplementary Figures S12, S13, S14). The heterogeneity observed in each of the funnel plots was very high (I^2^ > 95%), and formal tests of funnel plot asymmetry (Egger’s regression test and Begg’s test) were considered unreliable^29^.

## Discussion

This systematic review and meta-analysis pooled data from fifty-two case control studies to evaluate serum lipid alterations (TC, HDL, LDL, VLDL and TGs) in OPMD and OSCC compared to healthy controls. We observed consistent reductions across multiple lipid fractions in both OPMD and OSCC relative to controls. The magnitude of reduction tended to be greater in OSCC than in OPMD, a pattern consistent with a model in which lipid alterations are associated with disease stage rather than reflecting a temporal or causal relationship, particularly given the predominantly cross-sectional design of the included studies. Potential factors influencing lipid levels, such as statin therapy, diabetes, nutritional status, and systemic inflammation, may also contribute to the observed differences between cases and controls.

To contextualize these findings, several biological mechanisms reported in cancer metabolism research may be relevant, though they cannot be directly inferred from the included studies. Tumor cells depend on enhanced lipid turnover and upregulated lipogenic enzymes such as fatty acid synthase and acetyl-CoA carboxylase, facilitating de novo lipogenesis and lipid uptake^100^. Such metabolic reprogramming may reduce circulating cholesterol and triglyceride pools needed for membrane biogenesis and signalling^101^.

Furthermore, oxidative stress and lipid peroxidation are well documented in oral dysplasia and carcinoma, offering additional mechanisms for lipid depletion and altered HDL mediated antioxidant activity^102^. Moreover, systemic inflammation and hepatic metabolic changes associated with malignancy may contribute to altered LDL and VLDL levels^103,104^. These mechanistic considerations align with experimental and molecular evidence, although clinical cross-sectional data are insufficient to establish directional or explanatory links.

An additional consideration is the potential influence of nutritional status and cancer-related metabolic changes on serum lipid profiles. Malnutrition and cancer cachexia, both highly prevalent in patients with oral cancer are known to reduce circulating LDL, HDL and TGs through increased catabolism and altered hepatic synthesis^105–107^. Moreover, treatment modalities such as radiotherapy and chemotherapy can modify lipid metabolism via oxidative stress, systemic inflammation and hepatic effects, contributing to changes in HDL functionality and TGs turnover^108,109^. However, most studies did not consistently report the timing of blood collection (pre- vs. post-treatment), with the exception of one study^34^. In addition, nutritional status and detailed treatment history were not reported in most studies, limiting the ability to disentangle disease-related lipid alterations from confounding effects related to therapy or nutrition. A few studies examined tobacco or gutka use and serum lipid profiles in patients with oral cancer and precancerous conditions^53,60,61,65^, and comparisons between smokers and non-smokers^87,110^. However, the data were insufficient and heterogeneous to allow pooling or meta-analysis.

Future studies should incorporate standardized assessments of nutritional status, cachexia markers and treatment exposure to more accurately characterise lipid changes in OPMD and OSCC. In OPMD, including LK, OSMF and LP, most studies reported reduced serum TC, HDL and LDL compared with controls, consistent with the presence of chronic inflammation, oxidative stress and epithelial dysplasia that characterize these lesions^22,44^. The generally lower lipid levels in OPMD relative to controls, and the additional decrease observed in OSCC, support an association between lipid disturbance and increasing disease severity, although the direction of relationship is unknown.

Individual studies demonstrated heterogeneity across OPMD subtypes. Several investigations from India and China reported substantial reductions in TC, HDL, and LDL in OSMF, and LP compared with controls, consistent with increased oxidative stress and inflammation^22,25,59^.

In contrast, some studies found lipid levels in LK closer to control values or showing less pronounced reductions, suggesting that metabolic alterations vary by lesion type and underlying pathology^22^. A 2014 analysis of OSMF further indicated a stage dependent decline in multiple lipid fractions, linking more advanced fibrosis and epithelial atrophy with lager metabolic disturbances^80^. While lipid depletion is a frequent finding in OPMD, its magnitude and pattern differ across lesion categories.

Results concerning lipid differences across OSCC histopathological grades and clinical stages were heterogeneous. Some studies observed progressively decreasing lipid levels, particularly in poorly differentiated or advanced tumors, consistent with increased metabolic demand in aggressive disease^60,80^. Others found minimal or no significant grade or stage related differences^11,61,63,67^. A recent study suggested that VLDL and TGs may show the most pronounced declines in poorly differentiated OSCC, while other lipid fractions remained inconsistent^22^. This variability likely reflects methodological differences, limited sample sizes and insufficient control for confounding factors, precluding definitive conclusions regarding lipid profiles as indicators for tumor aggressiveness.

Adherence to PRISMA 2020 guidelines, dual independent screening and extraction, and random-effects meta-analysis strengthen the methodological robustness of this review. Including three comparison groups (OSCC vs. controls, OPMD vs. controls, and OSCC vs. OPMD) allowed for structured assessment of lipid changes across clinically distinct disease categories, providing a broad framework for interpreting lipid alterations in relation to disease status. However, several limitations must be considered when interpreting the findings. Heterogeneity between studies was high, likely reflecting variation in study populations, lipid assay methodologies, clinical classification of OPMD, and incomplete adjustment for key confounders such as age, sex, tobacco and alcohol exposure, metabolic status, and nutritional factors. The geographic concentration of evidence predominantly from India, may limit generalisability to other populations. Most included studies were small, single-centre, and cross-sectional, restricting the ability to determine temporality or causality. Although funnel plots suggested some degree of publication bias, the overall consistency in the direction of effects supports the reliability of the main patterns observed. The lack of longitudinal or treatment-stratified data prevents assessment of how lipid levels change over time or in response to therapy. Despite these limitations, the convergence of findings across many independent studies supports the robustness of the observed associations between reduced serum lipid levels and oral disease status.

## Conclusion

This systematic review and meta-analysis provide evidence of consistent reductions in serum lipid levels including TC, HDL, LDL, VLDL, and TGs in patients with both OPMD and OSCC compared with healthy controls. Greater reductions observed in OSCC relative to OPMD support an association between lipid alterations and disease stage, although causality cannot be established due to the cross-sectional nature of the included studies. The findings suggests that systemic lipid depletion may be an early and progressive feature in oral carcinogenesis, potentially influenced by enhanced lipid utilization, oxidative stress, and inflammatory processes. While these results highlight the potential utility of serum lipid profiling as a non-invasive biomarker for disease monitoring and risk stratification, interpretation should be tempered by substantial heterogeneity across studies, geographic concentration of the evidence, and incomplete control for confounding factors such as nutrition, lifestyle, metabolic comorbidities, and treatment status. Future research should prioritize prospective, longitudinal studies with standardized lipid assessment, careful adjustment for confounders, and inclusion of diverse populations to clarify the clinical and prognostic relevance of serum lipid alterations in OPMD and OSCC.

## Supplementary Information

Below is the link to the electronic supplementary material.

Supplementary Material 1

## Data Availability

In this systematic review and meta-analysis, all study-level data required to reproduce the analyses (means, standard deviations, and sample sizes) are already transparently reported in the forest plots; therefore, providing the extracted Excel files would represent only an alternative data format rather than additional raw data. Any further details are available from the corresponding author upon reasonable request.
