# Supplementary material for: Serum lipid alterations in oral squamous cell carcinoma and oral potentially malignant disorders: A systematic review and meta-analysis
Source: Sci Rep. 2026 Mar 31;16:10998. doi: 10.1038/s41598-026-46163-z (PMC13043762; doi:10.1038/s41598-026-46163-z)
Supplement: Supplementary file 1 — Supplementary Material 1 [file 41598_2026_46163_MOESM1_ESM.docx]

**Supplementary**

**List of Excluded Articles and Corresponding Reasons for Exclusion**

| **Author (Year)** | **Reason for exclusion** |
| --- | --- |
| Mehrotra et al (2009) [1] | Lack of control group data |
| Sharma et al (2013) [2] | Small sample size. No qualitative data for meta-analysis |
| Pantvaidya et al (2013) [3] | Editorial review, no quantitative data available |
| Sherubin et al (2013) [4] | Plasma lipid levels analysed across histopathological grades in OSCC but did not include a control group. |
| Bailwad et al (2014) [5] | Although the paper is on lipid profile of OSCC patients, No quantitative data available |
| Rawson et al (2015) [6] | No qualitative data for meta-analysis. |
| Baduni et al (2015) [7] | No qualitative data for meta-analysis |
| Subbulakshmi et al (2017) [8] | No qualitative data available for meta-analysis |
| Wang et al (2017) [9] | Clinical study, analyzed plasma lipidomics (glycerophospholipids, sphingolipids) but did not assess conventional serum lipids. |
| Rakheerathnam et al (2018) [10] | No quantitative data for meta-analysis |
| Rahman et al (2020) [11] | Clinical study focused on lysophosphatidic acid in plasma, not standard lipid profiles (TC, LDL, HDL, VLDL), making it irrelevant |
| Raychaudhuri et al (2020) [12] | Spectrum of OSCC in a tertiary care hospital, and does not give the lipids profile, ineligible study design |
| Sachdev et al (2020) [13] | No quantitative data for meta-analysis |
| Tantray et al (2020) [14] | No quantitative data for meta-analysis |
| Wang et al (2021) [15] | In vitro cell line study, as it analyzed lipid profiles in CAL-27 tongue cancer cells and normal oral keratinocytes. |
| Siddiq et al (2021) [16] | Lack of control group |
| Wilms et al (2021) [17] | Lacks quantitative data |
| Bhat et al (2022) [18] | No quantitative data for analysis |
| Jayanthiswari et al (2022) [19] | No control group. They compare pre and post serum profile of OPMD patients. |
| Khyalia (2022) [20] | Study on serum lipid profile among OSMF and OSCC and their comparative analysis, no control group was included. |
| Sangle et al (2023) [21] | No qualitative data for meta-analysis |
| Wang et al (2023) [22] | Clinical study, focused on broad metabolomic and lipidomic profiling rather than standard lipid parameters (TC, LDL, HDL, VLDL), and it did not specifically correlate these lipids with clinical outcomes |
| Ma et al (2023) [23] | Although the study includes lipid parameters (TC, HDL, Apo-B), it is based on GWAS data analysis rather than direct clinical measurement of blood lipid profiles, which do not meet inclusion criteria |
| Garg et al (2024) [24] | Compares serum lipid levels among OSCC, OPMD, and healthy controls; however, the exclusion criteria used in that study do not align with our study design. |
| Sharma et al (2024) [25] | Compares serum lipids in OSCC but, no quantitative data available for analysis |
| Ali et al (2025) [26] | Review article |
| Gonzalez et al (2025) [27] | Review article |
| Kumar et al (2025) [28] | No qualitative data available |
| Tyagi et al (2025) [29] | Compares serum lipids in head and neck cancers but does not specifically include OSCC |

**Table S1.** Excluded articles with reason for exclusion

**Summary of Included Articles on Serum Lipids and their Association with OSCC and OPMD**

| **Authors (years)** | **Study population Age & Sex** | **Population comparison** | **Geographic area** | **Sample collection** | **Lipids analysis** | **Main Outcomes** |
| --- | --- | --- | --- | --- | --- | --- |
| Patel et al (2004) [30] | HNC: 50 yrs, 142m, 42f, control: healthy close relatives | 184 OSCC, 153 OPMD, 52 controls | India | Blood sample | EC (units: mg/dl) | Significant decrease in TC, HDL, VLDL, and TGs in cancer and OPMD patients compared to controls, with greater reduction in OSCC |
| Nayak et al (2010) [31] | OPMD: 31.4±10.5 16m, 12f; control: 28±7.5 16m, 12f | 56 participants, 14 OSMF, 14 LP, 28 controls | India | Blood sample | EC (units: mg/dl) | Reduction in TC, HDL, and LDL levels in OPMD cases compared to age- and sex-matched healthy controls. |
| Lohe et al (2010) [32] | OSCC: 36 m & 34 f, age (41.99± 15.72); control: 40 m & 30 f, age (42.36 ± 14.28) | 70 OSCC, 70 controls | India | Blood sample | EC (units: mg/dl) | Decrease in serum lipid levels particularly TC and HDL in OSCC compared to control |
| Chawda et al (2011) [33] | 30 male; Age (50.66 years) | 30 subjects, 25 OSCC and 5 healthy controls | India | Blood sample | EC (units: mg/dl) | OSCC patients had lower lipids, TC, HDL than controls |
| Gupta et al (2011) [34] | LK: 42.27 yrs, 15m; OSMF: 29.87 yrs, 14m, 1f; LP: 36.40 yrs, 12m, 3f | 70 participants, 25 OSCC; 15 LK, 15 OSMF; 15 LP; 15 controls | India | Blood sample | EC (units: mg/dl) | Decrease in TC, HDL, and TGs in patients with OPMD compared to controls, indicating an inverse relationship with disease. Compared to OSCC patients, they showed relatively higher TC but lower HDL and TGs levels. |
| Kumar et al (2012) [35] | OSCC: 25m, 5f, age (34.9±8.44); oral LK 25m, 5f, age (45.23±12.18) control: 28m, 2f, age (38.26±11.61) | 90 participants: 30 OSCC, 30 oral LK, 30 controls | India | Blood sample | EC (units: mg/dl) | TC, HDL, and LDL were significantly lower in the OSCC group compared to controls |
| Anuradha et al (2013) [36] | OSCC: Mean age 45yrs, 18m, 5f; Control: 61m, 12f | 23 OSCC, 73 controls | India | Blood sample | EC (units: mg/dl) | Significant decrease in TC and LDL, while HDL was elevated compared to controls. TGs and VLDL were lower but not statistically significant, same trends in males and females. |
| Kumar et al (2013) [37] | OPMD: 49.5±9.3, 14m, 6f; Control: 33.4±8.3, 6m, 4f | 30 participants, 20 OSMF, 10 controls | India | Blood sample | EC (units: mg/dl) | Significant decrease in TC, LDL, and HDL compared to controls, reductions in VLDL and TGs were not statistically significant. |
| Singh et al (2013) [38] | OSCC: 51.07 yrs, 40m, 35f; control: age and sex matched | 75 participants, 50 OSCC, 25 controls | India | Blood sample | EC (units: mg/dl) | Significant decrease in TC, HDL, and TGs levels in the OSCC compared to controls. No significant differences observed across histological grades of OSCC. |
| Srinivas et al (2013) [39] | OSCC: 54.2±11.74, 7m, 3f; control: 48.5± 12.87, 6m, 4f | 20 participants, 10 OSCC, 10 controls | India | Blood sample | EC (units: mg/dl) | Between groups, LDL showed an overall significant difference. TC, TGs, HDL, and VLDL changes were mostly non-significant across the groups |
| Ajai et al (2014) [40] | OPMD: 33m, 12f (29.09yrs), sex matched control (29.09 yrs) | 90 participants, 45 OSMF 45 controls | India | Blood sample | EC (units:mg/dl) | Serum lipid levels were significantly lower in OSMF patients compared to controls |
| Banseria et al (2014) [41] | OSCC & Control: Age 41-60; 68 m, 32 f | 100 OSCC, 100 controls | India | Blood sample | EC (units: mg/dl) | Decrease in lipid profile components in OSCC patients |
| Ganavi et al (2014) [42] | OSCC: Age 53.6 yrs, 5m, 5f; LK: Age 51.2 yrs, 6m, 4f; control: age 40.3yrs, 3m,7f | 30 participants, 10 OSCC, 10 LK, 10 controls | India | Blood sample | EC (units: mg/dl) | OSCC had lower TC than controls, while LK had intermediate levels no statistical significance. A feasible prognostic tool in LK patients. |
| Garg et al (2014) [43] | OSCC: age 35–70 years, 17 m, 3 f. Control: age 30–72 years, 14 m, 6 f. | 20 OSCC and 20 controls | India | Blood sample | EC (units: mg/dl) | Inverse relationship between plasma lipid levels and disease presence. |
| Goel et al (2014) [44] | OPMD: 37.2 yrs, 51m, 9f; control: 43.5 yrs, 14m, 6f | 80 participants, 20 LK, 20 OSMF, 20 LP, 20 controls | India | Blood sample | EC (units: mg/dl) | Inverse relationship between serum lipid profile and OPMD. Significant reductions in TGs and VLDL were observed in LK and LP, no significant changes in OSMF patients. |
| Kamath et al (2014) [45] | OSCC: 58.5yrs, 14m, 14f; age and sex matched sex | 56 participants, 28 OSCC, 28 controls | India | Blood sample | EC (units: mg/dl) | TC, TGs, LDL, and VLDL were slightly elevated, while HDL was markedly reduced compared to controls. |
| Mahesh et al (2014) [46] | oral LK: mean age 52.6 years, m:f 4:1, controls: age 40.3 years, 3:2 ratio | 30 participants, 15 with oral LK and 15 controls | India | Blood sample | EC (units: mg/dl) | Oral LK showed lower TC, HDL, LDL, TGs, higher VLDL levels than controls |
| Mehta et al (2014) [47] | M 50, f 10 in each group; OPMD age (32.60 ±11.79), OSCC (50.10 ±7.66), control (below 65) | 60 OPMD, 60 OSCC, 60 controls. | India | Blood sample | EC (units: mg/dl) | OSCC patients showed decreased HDL, lower TC, TGs compared to controls |
| Neerupakam et al (2014) [48] | Age (40 – 70 years) | 30 individuals: 15 OSCC patients and 15 control subjects | India | Blood sample | EC (units: mg/dl) | OSCC patients showed lower TC, HDL, LDL, with elevated VLDL and reduced TGs, altering lipid profiles. |
| Radhakrishna et al (2014) [49] | OSMF: 20-65 yrs, 30m; control 20-65 yrs, 19m | 30 OSMF, 19 controls | India | Blood sample | EC (units: mg/dl) | Significant decrease in TC and TC:HDL ratios in OSMF compared to controls. No significant differences were found across OSMF histologic grades. |
| Sasankoti et al (2014) [50] | Age and sex matched | 95 participants, 35 OSCC, 30 OSMF, 30 controls | India | Blood sample | EC (units: mg/dl) | Mean lipid levels were highest in OSCC for all parameters except HDL. The lowest values were seen in OSMF patients for most variables, while LDL was lowest in controls. |
| Agarwal et al (2015) [51] | Mean age 41.4yrs; OSCC 19m, 6f; OSMF 20m, 5f; control 43m, 32f | 125 participants, 25 OSCC, 25, OSMF, 75 controls | India | Blood sample | EC (units: mg/dl) | Male predominance (69%) and found significantly lower serum lipid levels in OSMF and OSCC patients compared to controls. |
| Dongre et al (2015) [52] | Mean age OSCC: 48.85 ± 10.81 yrs; OSMF: 29.65 ± 7.63 yrs; control: 30.25 ± 10.59 yrs | 60 participants, 20 OSCC, 20 OSMF, 20 healthy controls | India | Blood sample | EC (units: mg/dl) | Lower serum lipid profile levels, particularly decreased HDL and Apoprotein A1, are significantly associated with OSMF and OSCC compared to controls. |
| Kanthem et al (2015) [53] | Age and sex matched | 50 OSMF, 50 controls | India | Blood sample | EC (units: mg/dl) | Significantly decreased TC, HDL, and LDL levels in OSMF compared to controls, with TC and HDL levels declining further as clinical stage progressed. All lipid parameters reduced with advancing histological grade. |
| Acharya et al (2016) [54] | OSCC: mean age 48.6 years, 74 m,16 f; control: age & sex matched | 120 participants, 90 OSCC and 30 controls | India | Blood sample | Photometric EC (units: mg/dl) | Significant decrease serum TC, HDL, and LDL levels in OSCC patients compared to controls. No differences across stage, grade, or lymph node status, suggesting lipid reduction occurs during OSCC development and progression. |
| Li et al (2016) [55] | OSCC: Age mean 60 years, 74 m, 62 f, control: age and sex matched | 136 with OSCC control 136 | China | Blood sample | EC and Immunoturbidimetry (units: mmol/l) | Lower levels of TC, LDL, apo A, and B, but higher levels of lipoprotein |
| Mujoo et al (2016) [56] | OSMF: 29.60 ± 9.92, 26m, 4f; LK: 38.27 ± 10.82, 28m, 2f; Control: 37.63 ± 7.19, 27m, 3f | 90 participants, 30 OSMF, 30 LK, 30 controls | India | Blood sample | EC (units: mg/dl) | Serum TC and TGs were significantly reduced in OPMD cases compared to controls, while other lipid and glucose parameters showed no significant difference. |
| Poorey et al (2016) [57] | OSCC: age 41 – 60 years, 68 m, 32 f; control: age & sex matched | 100 with OSCC & 50 controls. | India | Blood sample | EC (units: mg/dl) | OSCC showed reduced TC, TGs, HDL, lower levels in tobacco users compared to controls |
| Reddy et al (2016) [58] | 40-70 yrs, mostly male | 25 OSCC, 15 controls | India | Blood sample | EC (units: mg/dl) | OSCC patients had increased LDL but reduced TC, TGs, VLDL, and HDL compared to controls |
| Vyas et al (2016) [59] | Mean 19 – 78 yrs; OSCC: 17m, 8f; control: 62m, 38f | 125 participants, 25 OSCC, 100 controls | India | Blood sample | EC (units: mg/dl) | Significant declining trend in TC, LDL, VLDL, HDL, and TGs in OSCC compared to control. The greatest reduction seen in TGs (22%). |
| Bhagat et al (2017) [60] | Age: 31- 60 yrs; OSCC: 8m, 2f; OPMD: 18m, 2f; control 14m, 6f | 10 OSCC, 20 OPMD (10 LK, 10 OSMF), 20 controls | India | Blood sample | EC (units: mg/dl) | OSCC patients showed significantly decreased TC and LDL levels, while OPMD had significantly increased HDL levels compared to controls. |
| Swamy et al (2017) [61] | OPMD: 45.5±11.7, 30m, 20f; control: 45.2±10.3, 30m, 20f | 100 participants, 50 OSMF, 50 healthy controls | India | Blood sample | EC (units: mg/dl) | Significant reduction in serum TC, TGs, LDL, VLDL, and HDL was observed in patients with OSMF compared to healthy controls |
| Anand et al (2018) [62] | Age and sex matched | 30 OSCC, 30 OSMF, 30 controls | India | Blood sample | EC (units: mg/dl) | Study showed a significant reduction in serum lipid levels in patients with OSCC and OSMF compared to healthy controls |
| Anusha et al (2018) [63] | Age 20 – 50 yrs, age and sex matched control | 150 participants, 120 OPMD, 30 controls | India | Blood sample | EC (units:mg/dl) | Significant reduction in cholesterol levels in OSMF compared to controls, with greater decreases as disease severity progressed |
| Yashveer et al (2018) [64] | OPMD: mean age 32.1 years, m:f 1.72:1; control: mean age 32.8 years m:f 1.14:1 | 30 OSMF, and 30 controls | India | Blood sample | Spectrophotometry (units: mg/dl) | OPMD patients had lower TC, TGs, HDL, LDL, VLDL than controls. |
| Vijay et al (2019) [65] | OPMD: mean age 44.15 yrs, control: mean age 40.4 yrs, | 50 participants, 20 LK, 30 control | India | Blood sample | EC (units: mg/dl) | TGs, LDL, and VLDL higher in males, while TC and HDL were higher in females but not significant. LK patients showed significantly reduced serum lipid levels. |
| Vyas et al (2019) [66] | Age: 15-55 yrs, age and sex matched control | 160 participants, 20 OSCC, 20 OSCF, 20 LK, 100 control | India | Blood sample | EC (units: mg/dl) | Greatest reduction in serum lipids observed in OSCC. While OSMF and LK showed similar lipid profiles, and significantly lower than controls. |
| Ramesh et al (2020) [67] | OSMF: mean age 34.65 yrs, control: 31.10 yrs | 40 participants, 20 OSMF, 20 controls | India | Blood sample | EC (units: mg/dl) | Significantly lower serum lipid levels, hemoglobin, RBCs, and platelets in OSMF patients compared to controls. ESR and WBC counts were elevated in the OSMF group. |
| Agrawal et al (2021) [68] | OSCC: mean age 50.97±9.18, 26m, 4f; LK 46.17±10.26, 30m, 0f; OSMF 51.73±8.81, 29m, 1f; control 50.70±9.19, 27m, 3f | 120 participants, in each group | India | Blood sample | EC (units: mg/dl) | Significantly reduced TC, TGs, LDL, HDL, and VLDL levels in OSCC, OSMF, and LK patients compared to healthy controls |
| Siddique et al (2021) [69] | OPMD: 32-68 yrs, 20m, 10f; control: 28-57 yrs, 20m, 10f | 30 OSMF, 30 controls | Pakistan | Blood sample | EC (units: mg/dl) | Serum lipid levels were markedly lower in OPMD compared to controls. |
| Singh et al (2021) [70] | OSCC: 44.3yrs, 34m, 18f; LK: 43.4 yrs, 19m, 7f; OSMF: 35.2 yrs, 19m, 7f; control: 29.7 yrs, 14m, 11f | 129 participants, 52 OSCC, 26 LK, 26 OSMF, 25 controls | India | Blood sample | EC (units: mg/dl) | No significant intergroup difference in serum and salivary TC and HDL levels, while salivary TGs showed a significant variation among groups |
| Alazzawi et al (2022) [71] | OSCC: age 63 years, 11 m, 11 f; control: mean age 62.4yrs, 8 f, 14 m | 44 participants, 22 with OSCC and 22 healthy controls | Iran | Blood sample | EC and ELISA (units: mg/dl) | OSCC patients showed reduced serum lipid profile and Apolipoprotein (Apo)-C-1 concentration compared to controls. |
| Chowdhury et al (2022) [72] | OSCC: 30-70, OPMD: 28 – 70; Control: 28-70yrs. | 225 participants, 75 OSCC, 75 OSMF, 75 controls | India | Blood sample | EC (units: mg/dl) | HDL and VLDL levels were significantly lower in the OSCC group compared to controls, while LDL showed no significant difference. |
| Khyalia et al (2022) [73] | OSCC: 30 -65 yrs, 51.13±8.56, 35m, 10f; and Control: 49.22±9.21, 35m, 10f | 90 participants, 45 OSCC; 45 healthy controls | India | Blood sample | EC (units: mg/dl) | OSCC patients showed significantly altered TC, HDL, and LDL levels, compared to healthy controls. |
| Sachin et al (2022) [74] | OPMD: 83m, 17f; control: 4m, 16f; Age matched | 100 participants with OPMD, 20 controls | India | Blood sample | EC (units: mg/dl) | Significantly lower serum HDL levels in females with OPMD, with LK showing reduced TC, HDL, and VLDL. |
| Sai et al (2022) [75] | OSCC: age 51.2yrs, 24m, 6f; OPMD: age 44.5 yrs, 21m, 9f | 90 participants, 30 OSCC, 30 OPMD, 30 controls | India | Blood sample | EC (units: mg/dl) | Reduction in serum TC, LDL, and CHO/HDL ratio was noted in OPMD and OSCC groups compared to controls. Poorly differentiated OSCC showed a decrease VLDL and TGs. |
| Vikram et al (2022) [76] | age and sex-matched, apparently healthy volunteers | 100 participants, 50 moderate OSMF, 50 controls | India | Blood sample | EC (units: mg/dl) | Patients with moderate OSMF showed significantly reduced serum levels of TC, TGs, HDL, LDL, and VLDL compared to controls |
| Adnan et al (2023) [77] | OSCC: mean age 48 yrs, 35m, 15f; control: age and sex matched | 75 participants, 50 OSCC, 25 controls | Syria | Blood sample | EC (units: mg/dl) | Significant reduction in TC, HDL, LDL, VLDL, and TGs levels in the OSCC group compared to controls |
| Rashmi et al (2023) [78] | OSCC: 51.42, 34m, 16f; OPMD: 40.46, 37m, 13f; Control: 41.36, 29m, 21f | 150 participants, 50 OSCC, 50 OPMD, 50 controls | India | Blood sample | EC (units: mg/dl) | Compared to controls, both OSCC and OPMD showed decreased TC, HDL, LDL, and VLDL levels. TGs, no significant change. |
| Qian et al (2023) [79] | OPMD: 51±13 years, 42m, 103 f; OSCC: Age 61±10 years, 117 m, 70 f; control: Age 52±11, 116m, 84f | 145 OPMD, 187 OSCC, and 200 control. | China | Blood sample | EC and Immunoturbidimetry (units: mmol/L) | High HDL and Apo-A linked to OPMD, with age, sex influencing lipid profile patterns |
| Varshney et al (2023) [80] | 82 patients with histopathologically diagnosed OSCC, 38 age & sex matched healthy control | 120 participants, 82 OSCC, 38 controls | India | Blood sample | EC (units: mg/dl) | TC, LDL, VLDL, HDL, and TGs showed statistically significant differences between OSCC and controls. Significant decrease in TC and HDL with loss of differentiation. |
| Chandra et al (2025) [81] | OSCC: 50.21 ± 11.70 yrs, 61m, 14f; OPMD: 40.55 ±13.71 yrs, 65m, 10f; Control: age & sex matched | 225 participants, 75 OPMD, 75 OSCC, 75 controls | India | Blood sample | EC (units: mg/dl) | OPMD and OSCC patients showed significantly elevated inflammatory and platelet indices with reduced RBC, hemoglobin, platelet count, and HDL compared to controls. |

**Table S2.** Summary of included articles exploring the association of serum lipids in OSCC and OPMD.

Abbreviations: HDL, high-density lipoproteins; LDL, low-density lipoproteins; VLDL, very low-density lipoproteins; TGs, triglycerides; TC, total cholesterol; OSCC, oral squamous cell carcinoma; OPMD, oral potentially malignant disorder; OSMF, oral submucous fibrosis; L, Leukoplakia; LP, lichen planus; EC, Electrochromatography.

**Study-wise Risk of Bias (RoB) Assessment and Reasons**

| **Authors (years)** | **Rank** | **Reasons** |
| --- | --- | --- |
| Patel et al (2004) [30] | Low | Potential selection bias from hospital-based controls and limited adjustment for confounders. Other domains, including diagnostic verification and laboratory methods, demonstrated a low RoB. |
| Nayak et al (2010) [31] | Low | Potential selection bias from limited control description and lack of confounder adjustment, despite standardized diagnostic and analytical methods. |
| Lohe et al (2010) [32] | Low | Single-center recruitment, limited blinding, small subgroups, and uncontrolled confounders, though standardized assays and well-defined groups are strengths. |
| Chawda et al (2011) [33] | Serious | Strengths include standardized assays and clearly defined patient groups. Limitations are small, unbalanced sample, single-center recruitment, limited blinding, and unadjusted confounders. |
| Gupta et al (2011) [34] | Moderate | Strengths include standardized assays, histopathological confirmation, and age- and sex-matched controls. Limitations include small, hospital-based sample, potential lack of blinding, and unadjusted confounders. |
| Kumar et al (2012) [35] | Low | Strengths include prospective design, independent histopathology grading, balanced group sizes, and standardized assays. Limitations include single-center recruitment, male-dominated sample, some unblinded outcome assessment, and small subgroup sizes for some analyses. |
| Anuradha et al (2013) [36] | Low | Strengths include standardized biochemical assays and histopathological confirmation of cases. Limitations include single-center recruitment, gender imbalance in controls, small female subgroup, lack of explicit blinding, and unadjusted confounders. |
| Kumar et al (2013) [37] | Serious | Strengths include histopathological confirmation and standardized lipid measurements. Limitations are small and unbalanced sample, single-center recruitment, lack of blinding, and unadjusted confounders |
| Singh et al (2013) [38] | Moderate | Strengths include histopathological confirmation, standardized lipid measurement, and analysis considering tobacco habits. Limitations include single-center recruitment, smaller control group, lack of blinding, and unadjusted confounders. |
| Srinivas et al (2013) [39] | Serious | Strengths include standardized biochemical assays and histopathological confirmation for cases. Limitations include very small sample size, single-center recruitment, unbalanced sex distribution, lack of blinding, and unadjusted confounders. |
| Ajai et al (2014) [40] | Moderate | Strengths being moderate sample size, age- and sex-matched controls, standardized biochemical assays, histopathological confirmation of cases. Limitations are Single-center study, possible selection bias for controls, no explicit blinding in lab assays, some unmeasured confounders (diet, other habits) may influence lipid levels. |
| Banseria et al (2014) [41] | Low | Strengths are large sample size, histopathological confirmation, age- and sex-matched controls, standardized biochemical methods, exclusion of major comorbidities. Limitations are single-center study, potential selection bias for controls, no explicit blinding of lab personnel, residual confounding possible. |
| Ganavi et al (2014) [42] | Serious | Strengths are standardized laboratory methods, fasting blood samples, inclusion/exclusion criteria clear. Limitations are very small sample size, potential sex imbalance, unclear control selection, minimal control for confounders, no blinding mentioned. These factors collectively raise the RoB substantially. |
| Garg et al (2014) [43] | Serious | Strengths are standardized blood collection and laboratory analysis, clear inclusion/exclusion criteria. Limitations are small sample size, potential selection and confounding bias, slight sex imbalance, and no mention of blinding. |
| Goel et al (2014) [44] | Serious | Strengths are standardized measurement, histological confirmation, fasting blood samples. Limitations are small sample, sex imbalance, unclear matching/randomization, lack of blinding, and confounders not controlled. |
| Kamath et al (2014) [45] | Moderate | Clear inclusion/exclusion criteria, matched controls, standardized assays, internal quality control, and histological confirmation. Limitations are small sample size, convenience sampling, lack of blinding, and unadjusted confounding (notably tobacco use). |
| Mahesh et al (2014) [46] | Serious | Clear inclusion/exclusion criteria; use of automated analyzer; histopathological confirmation of LK. Weaknesses are poorly matched controls, lack of confounder adjustment (especially tobacco and alcohol), absence of blinding, small sample, and no stated QC/calibration procedures. |
| Mehta et al (2014) [47] | Low | Reasonable sample size, equal group distribution, objective lab methods, ethical clearance, and explicit inclusion/exclusion criteria. Limitations are lack of true randomization transparency, absence of blinding, unadjusted confounders (age and habits), and no mention of internal QC procedures. |
| Neerupakam et al (2014) [48] | Moderate | Ethical compliance, objective lab assays, standardized collection, clear exclusion of comorbidities. Limitations are small sample, unclear “randomization,” unmatched control group (especially for tobacco), no confounder control, and lack of blinding. |
| Radhakrishna et al (2014) [49] | Moderate | Strength includes Standardized sample handling, validated assays, objective measurements, clear inclusion/ exclusion criteria. Limitations are small sample, non-randomized design, all-male OSF group, habit mismatch with controls, lack of blinding, and no control for confounders like areca/tobacco use. |
| Sasankoti et al (2014) [50] | Moderate | Strengths are standardized fasting protocols, validated biochemical assays, clear data reporting. Limitations are non-random sampling, lack of blinding, no control for major confounders (tobacco, diet), small subgroups, and limited generalizability. |
| Agarwal et al (2015) [51] | Low | Recruitment from two sites and stratified grouping strengthen validity, but convenience sampling, unequal group sizes, and potential lack of blinding introduce moderate selection and detection bias. |
| Dongre et al (2015) [52] | Moderate | Single-center, and lack of randomization or blinding increase selection and detection bias. Although large sample size, standardized biochemical methods and statistical analyses were used, limited representativeness and unclear control selection reduce external validity. |
| Kanthem et al (2015) [53] | Low | Clear inclusion criteria, histopathological confirmation, and standardized biochemical assays, strengthen internal validity. However, single-center recruitment, convenience sampling, and lack of blinding or adjustment for confounders (e.g., diet, comorbidities) introduce moderate selection and detection bias. |
| Acharya et al (2016) [54] | Low | Reasonable methodological rigor with clear diagnostic and analytic procedures but retains moderate selection and confounding bias due to its non-randomized, hospital-based design and lack of blinding. |
| Li et al (2016) [55] | Low | Methodologically strong, with careful matching, standardized measurements, and robust statistical analysis. Minor limitations include hospital-based sampling and lack of explicit blinding, but these do not meaningfully compromise the validity of the findings |
| Mujoo et al (2016) [56] | Moderate | Methodologically acceptable, with matched controls and standard assays, but small sample size, hospital-based design, and limited control of confounders elevate RoB to moderate. Findings should be interpreted cautiously. |
| Poorey et al (2016) [57] | Low | Well-conducted study with objective outcome measurement, but some concern exists for selection bias due to restrictive exclusions and single-center recruitment. |
| Reddy et al (2016) [58] | Moderate | Main concern is selection bias due to small, unbalanced groups and convenience sampling. Objective nature of lipid measurements limits outcome bias, and verification and reference test biases are low. |
| Vyas et al (2016) [59] | Moderate | Main source of bias is selection bias due to small, unbalanced groups, single-center recruitment, and convenience sampling. Measurement of lipid outcomes is standardized, minimizing outcome and verification bias. |
| Bhagat et al (2017) [60] | Serious | The combination of non-random selection, inclusion bias from pre-existing test availability, and possible diagnostic and measurement inconsistencies indicate a serious RoB that may affect the reliability and generalizability of the findings. |
| Swamy et al (2017) [61] | Low | Main source of bias is selection bias due to small, single-center sample and likely convenience sampling, though age- and sex-matching reduces confounding. Laboratory outcomes are objective, minimizing verification and outcome bias. |
| Anand et al (2018) [62] | Low | Main concern is selection bias due to small, single-center, convenience-sampled groups. Laboratory measurements are objective, minimizing verification and outcome bias. |
| Anusha et al (2018) [63] | Moderate | Main concern is selection bias due to single-center recruitment, male-only participants, non-random sampling, and a large imbalance between case and control groups. Laboratory measurements are reliable and objective, minimizing other biases. |
| Yashveer et al (2018) [64] | Low | Main source of bias is selection bias due to single-center recruitment, small sample size, and non-random selection, but the study used objective laboratory measurements and matched controls, reducing other biases. |
| Vijay et al (2019) [65] | Moderate | Small sample size, non-randomized selection, and potential minor reporting inconsistencies. The objective nature of lipid measurements reduces bias in the outcomes. |
| Vyas et al (2019) [66] | Low | Non-randomized, convenience-based sampling, unequal group sizes, and minor reporting inconsistencies. The objective nature of serum lipid measurement reduces the likelihood of serious bias in outcome assessment. |
| Ramesh et al (2020) [67] | Low | Small sample size and single-center recruitment. Matching and objective measurements help reduce bias, but the limited population and short study duration may affect reliability and generalizability |
| Agrawal et al (2021) [68] | Low | The study used sound biochemical, statistical methods and large sample size, non-random hospital-based recruitment, and unadjusted confounding factors (especially lifestyle habits). Consequently, while the internal validity is fair, external validity and causal inference are limited. |
| Siddique et al (2021) [69] | Low | **Small sample size and single-center recruitment**. Matching and standardized measurements mitigate bias, but generalizability is limited. |
| Singh et al (2021) [70] | Low | Unequal group sizes, small control group, and limited detail on random selection. Biochemical measurements are objective, minimizing measurement bias, and outcomes were fully reported. |
| Alazzawi et al (2022) [71] | Low | Small sample size, potential selection bias, and lack of explicit blinding. Objective laboratory measurements and complete reporting reduce other biases. |
| Chowdhury et al (2022) [72] | Low | Risk of bias due to potential selection bias, limited description of laboratory methods, and lack of blinding. Large sample size and detailed reporting of outcomes and inclusion of all participants reduce other biases. |
| Khyalia et al (2022) [73] | Moderate | Selection bias due to recruiting patients from a single oncology outpatient department and excluding several conditions, and outcome/measurement bias because lipid levels were assessed using a single method without blinding, which may influence the reliability of comparisons between groups. |
| Sachin et al (2022) [74] | Moderate | Unbalanced and very small control groups, non-randomized sampling, and inconsistent reporting. Measurement methods are fairly standard, but lack of blinding and small volumes add moderate risk. The results should be interpreted with caution. |
| Sai et al (2022) [75] | Low | Strengths include well-defined inclusion/ exclusion criteria, equal-sized groups, and standard biochemical measurements. Limitations are small sample sizes in subgroups, lack of randomization, absence of blinding, and potential confounding from heterogeneous case groups. |
| Vikram et al (2022) [76] | Low | Strengths include clearly defined inclusion/exclusion criteria, matched controls, and standardized biochemical measurement. Limitations include lack of randomization, single-gender sample, and no mention of blinding. |
| Adnan et al (2023) [77] | Moderate | Selection bias due to small and unmatched control group, and possible confounding factors (e.g., smoking, diet, BMI) not fully addressed. Laboratory measurements are reliable, which reduces measurement bias. |
| Rashmi et al (2023) [78] | Low | Potential residual confounding bias (diet, lifestyle) and lack of blinding. The study design, matched groups, objective outcomes, and complete reporting reduce most other sources of bias. |
| Qian et al (2023) [79] | Low | Potential selection bias from the choice of controls and unmeasured confounding (lifestyle factors). Measurement and reporting biases are low due to objective biochemical measures and thorough statistical reporting. |
| Varshney et al (2023) [80] | Low | Good methodological rigor in recruitment, data collection, and laboratory analysis, but lacks adjustment for major lifestyle and nutritional confounders. Its hospital-based design and modest sample size also limit generalizability. |
| Chandra et al (2025) [81] | Low | There was potential selection bias due to the use of hospital-based controls. All other domains, including diagnostic verification, laboratory methods, and adjustment for confounders showed a low RoB. |

**Table S3.** Risk of Bias; RoB assessment for each included study with reason

**Sensitivity Analyses (Leave-One-Out Forest Plot) for OSCC and Healthy Controls**

The dots demonstrate the mean difference between OSCC and control groups. The horizontal lines and parentheses demonstrate the 95% CI. The vertical line demonstrate the overall SMD. P-values indicate the significance of the effect estimate after removal of each study.

**Figure S1.** Leave-one-out forest plot between oral squamous cell carcinoma; OSCC and healthy controls for total cholestrol; TC, confidence interval; CI, standardized mean difference; SMD.

**Figure S2.** Leave-one-out forest plot between oral squamous cell carcinoma; OSCC and healthy controls for (a) High-density lipoproteins; HDL, (b) Low-density lipoproteins; LDL, (c) Very low-density lipoproteins; VLDL, and (d) Triglycerides; TGs, confidence interval; CI, standardized mean difference; SMD.

**Leave-One-Out Sensitivity Analysis for OPMD and Healthy Controls**

**Figure S3.** Leave-one-out forest plot between oral potentially malignant disorder; OPMD and healthy controls for total cholestrol; TC, confidence interval; CI, standardized mean difference; SMD.

**Figure S4.** Leave-one-out forest plot between oral potentially malignant disorder; OPMD and healthy controls for (a) High-density lipoproteins; HDL, (b) Low-density lipoproteins; LDL, (c) Very low-density lipoproteins; VLDL, and (d) Triglycerides; TGs, confidence interval; CI, standardized mean difference; SMD.

**Leave-One-Out Sensitivity Analysis for OSCC and OPMD**

**Figure S5.** Leave-one-out forest plot between oral squamous cell carcinoma; OSCC and oral potentially malignant disorder; OPMD for total cholestrol; TC, confidence interval; CI, standardized mean difference; SMD.

**Figure S6.** Leave-one-out forest plot between oral squamous cell carcinoma; OSCC and oral potentially malignant disorder; OPMD for (a) High-density lipoproteins; HDL, (b) Low-density lipoproteins; LDL, (c) Very low-density lipoproteins; VLDL, and (d) Triglycerides; TGs, confidence interval; CI, standardized mean difference; SMD.

**Meta-Regression Analysis**

The meta-regression results across the OSCC, OPMD, and healthy controls status for each lipid has been plotted. We see that the study points are scattered vertically across almost the entire range of effect sizes for similar OPMD lipid levels suggesting no systematic relationship. The blue regression line is almost horizontal with a broad CI, consistent with R^2^ = 0, indicating that the meta-regression model provides no improvement over a standard random-effects meta-anlaysis model used in the rest of the manuscript. All meta-regression plots show high heterogeneity as seen through wide CI and scattered points. LDL alone shows a downward trend suggesting tumor lipid utilization and altered lipid metabolism during cancer progression, which may be due to one strong outlier (very negative SMD ≈ −12).

**Figure S7.** Meta-regression plot comparing oral squamous cell carcinoma; OSCC, oral potentially malignant disorder; OPMD, and healthy controls for total cholestrol; TC; x-axis displays the OPMD mean and y-axis shows the effect size (SMD) of OSCC relative to healthy controls.

**Figure S8.** Meta-regression plot comparing oral squamous cell carcinoma; OSCC, oral potentially malignant disorder; OPMD, and healthy controls for high-density lipoproteins; HDL; x-axis displays the OPMD mean and y-axis shows the effect size (SMD) of OSCC relative to healthy controls.

**Figure S9.** Meta-regression plot comparing oral squamous cell carcinoma; OSCC, oral potentially malignant disorder; OPMD, and healthy controls for low-density lipoproteins; LDL; x-axis displays the OPMD mean and y-axis shows the effect size (SMD) of OSCC relative to healthy controls.

**Figure S10.** Meta-regression plot comparing oral squamous cell carcinoma; OSCC, oral potentially malignant disorder; OPMD, and healthy controls for very low-density lipoproteins; VLDL; x-axis displays the OPMD mean and y-axis shows the effect size (SMD) of OSCC relative to healthy controls.

**Figure S11.** Meta-regression plot comparing oral squamous cell carcinoma; OSCC, oral potentially malignant disorder; OPMD, and healthy controls for triglycerides; TGs; x-axis displays the OPMD mean and y-axis shows the effect size (SMD) of OSCC relative to healthy controls.

**Visualization of Publication Bias: Funnel Plot for HDL, LDL, VLDL, and TGs**

**Figure S12.** Funnel plot between Oral squamous cell carcinoma; OSCC and healthy controls for a) High-density lipoproteins; HDL, b) Low-density lipoproteins; LDL, c) Very low-density lipoproteins; VLDL, and d) Triglycerides; TGs.

**Figure S13.** Funnel plot between oral potentially malignant disorder; OPMD and healthy controls for a) High-density lipoproteins; HDL, b) Low-density lipoproteins; LDL, c) Very low-density lipoproteins; VLDL, and d) Triglycerides; TGs.

**Figure S14.** Funnel plot between Oral squamous cell carcinoma, OSCC and oral potentially malignant disorder; OPMD for a) High-density lipoproteins; HDL, b) Low-density lipoproteins; LDL, c) Very low-density lipoproteins; VLDL, and d) Triglycerides; TGs.

**References**

1. Mehrotra, R., et al., Lipid profile in oral submucous fibrosis*.* Lipids in health and disease, **8**(1): p. 29; (2009).

2. Sharma, G., Das, D., Mukherjee, J. and Purandare, B., Lipid profile in oral submucous fibrosis patients in India-A pilot study*.* Indian J basic appl med res, **7**: p. 790-796; (2013).

3. Pantvaidya, G.H. and Katna, R., Oral submucous fibrosis and plasma lipid profile*.* South Asian journal of cancer, **2**(3): p. 145; (2013).

4. Sherubin, E., Kannan, S.K., Kumar, D. and Joseph, I., Estimation of plasma lipids and its significance on histopathological grades in oral cancer: Prognostic significance an original research*.* Journal of oral and maxillofacial pathology : JOMFP, **17**: p. 4-9; <https://doi.org/10.4103/0973-029X.110685> (2013).

5. Bailwad, S.A., et al., Alterations in serum lipid profile patterns in oral cancer: Correlation with histological grading and tobacco abuse*.* Oral health dent manag, **13**(3): p. 573-579; (2014).

6. Rawson, K., et al., Serum lipid profile as a prognostic marker in oral submucous fibrosis*.* Journal of Indian academy of oral medicine and radiology, **27**(4): p. 544-548; (2015).

7. Baduni, A., et al., Alterations in plasma lipid profile patterns in leukoplakia and oral submucous fibrosis-a pilot study*.* TC, **114**(128.57): p. 157-85; (2015).

8. Subbulakshmi, A.C., Mohan, N., Thiruneervannan, R. and Naveen, S., Comparative evaluation of serum lipid profile in patients with oral submucous fibrosis and oral squamous cell carcinoma with that of control subjects: A case control study*.* Journal of pharmacy & bioallied sciences, **9**(Suppl 1): p. S191; (2017).

9. Wang, L., et al., Plasma lipid profiling and diagnostic biomarkers for oral squamous cell carcinoma*.* Oncotarget, **8**(54): p. 92324; (2017).

10. Rakheerathnam, K.K., Chandran, C.R., Bhagavatham, M. and Devaraj, S.N., Role of trace elements and lipid Profile as markers of oral submucous fibrosis in Chennai population*.* IOSR Journal of dental and medical sciences, **17**(5): p. 74-81; (2018).

11. Rahman, M.A., et al., Profiling lysophosphatidic acid levels in plasma from head and neck cancer patients*.* PeerJ, **8**: p. e9304; (2020).

12. Raychaudhuri, S., et al., Spectrum of oral cancers in a tertiary care hospital in industrial belt of Haryana, India*.* Indian J pathol oncol, **7**(2): p. 253-8; (2020).

13. Sachdev, R., Garg, K., Singh, G. and Mehrotra, V., A comparative study to assess the independency of lipid profile and blood sugar levels as a diagnostic marker in oral cancer and precancerous disorders*.* Indian journal of dental sciences, **12**(4): p. 187-191; (2020).

14. Tantray, S., Sharma, S., Jhamb, P.A., Gupta, M. and Prabhat, K., Assessment of lipid profile in oral submucous fibrosis and oral squamous cell carcinoma*.* IJRA, **7**: p. 908-25; (2020).

15. Wang, X.-y., Zhang, T., Guan, W.-q., Li, H.-z. and Lin, L., A Study of the lipidomic profiles of the CAL-27 and HOK cell lines using EMS spectra*.* Frontiers in oncology, **11**: p. 771337; (2021).

16. Siddiq, M., Shakeel, J., Haider, S.M. and Siddiq, S., Mean lipid profile among patients suffering from oral sub-mucous fibrosis reporting in a tertiary care hospital of Karachi, Pakistan: A descriptive study*.* Annals of punjab medical college, **15**(1): p. 12-16; (2021).

17. Wilms, T., et al., High levels of low-density lipoproteins correlate with improved survival in patients with squamous cell carcinoma of the head and neck*.* Biomedicines, **9**: p. 506; <https://doi.org/10.3390/biomedicines9050506> (2021).

18. Bhat, D., Ahmad, D. and Chalkoo, A., Assessment of serum lipid profile in patients with oral potentially malignant disorders*.* International journal of applied dental sciences, **8**: p. 27-29; <https://doi.org/10.22271/oral.2022.v8.i4a.1621> (2022).

19. Jayanthiswari, K., Manoharan, G.V.M.G. and Muthukumar, S., Serum lipid profile and C-reactive protein as prognostic markers in oral submucous fibrosis*.* Journal of Indian academy of oral medicine and radiology, **34**(2): p. 166-170; (2022).

20. Khyalia, S., Study of serum lipid profile in patients with oral submucous fibrosis and oral squamous cell carcinoma and their comparative analysis*.* Journal of research in applied and basic medical sciences, **8**(2): p. 90-96; (2022).

21. Sangle, V.A., et al., Alterations in serum lipid profile in oral cancer and oral submucous fibrosis patients: A clinicopathological study*.* Oral & maxillofacial pathology journal, **14**(2); (2023).

22. Wang, Z., et al., Combined metabolomic and lipidomic analysis uncovers metabolic profile and biomarkers for papillary thyroid carcinoma*.* Scientific reports, **13**(1): p. 17666; (2023).

23. Ma, Q., Li, Y., An, L., Guo, L. and Liu, X., Assessment of causal association between differentiated thyroid cancer and disordered serum lipid profile: a Mendelian randomization study*.* Frontiers in endocrinology, **14**: p. 1291445; (2023).

24. Garg, K., Lipid profile in oral potentially malignant disorders and oral squamous cell carcinoma*.* Journal of research and advancement in dentistry, **15**: p. 1-3; <https://doi.org/10.53064/jrad.2024.15.5.546> (2024).

25. Sharma, A. and Tyagi, S., Serum lipid profile in patients with oral squamous cell carcinoma*.* International journal of life sciences, biotechnology and pharma research, **13**(3): p. 711-714; (2024).

26. Ali, A., et al., Immune microenvironment in oral potentially malignant disorders and oral cancer: A narrative review*.* International journal of molecular sciences, **26**(14): p. 6650; (2025).

27. González-Ruiz, I., Samayoa-Descamps, V., Guagua-Cortez, K.A., González-Moles, M.Á. and Ramos-García, P., Hallmarks of cancer expression in oral leukoplakia: A scoping review of systematic reviews and meta-analyses*.* Cancers, **17**(15): p. 2427; (2025).

28. Kumar, R., et al., Evaluation of atherogenic index of plasma and lipid ratios in patients with head and neck squamous cell carcinoma in north Indian study group*.* International journal of pharmacy research & technology (IJPRT), **15**(2): p. 871-876; (2025).

29. Tyagi, A. and Goel, S., Evaluation of diagnostic role of serum lipid profile in head and neck malignancy*.* European journal of cardiovascular medicine, **15**: p. 941-946; (2025).

30. Patel, P.S., et al., Alterations in plasma lipid profile patterns in head and neck cancer and oral precancerous conditions*.* Indian journal of cancer, **41**(1): p. 25-31; (2004).

31. Nayak, P., Nayak, S. and Darafsh, M.D., Alteration in plasma lipid profile in precancerous conditions*.* Journal of Nepal dental association, **11**(1): p. 40-45; (2010).

32. Lohe, V.K., Degwekar, S.S., Bhowate, R.R., Kadu, R.P. and Dangore, S.B., Evaluation of correlation of serum lipid profile in patients with oral cancer and precancer and its association with tobacco abuse*.* Journal of oral pathology & medicine, **39**(2): p. 141-148; (2010).

33. Chawda, J.G., Jain, S.S., Patel, H.R., Chaduvula, N. and Patel, K., The relationship between serum lipid levels and the risk of oral cancer*.* Indian journal of medical and paediatric oncology, **32**(01): p. 34-37; (2011).

34. Gupta, S. and Gupta, S., Alterations in serum lipid profile patterns in oral cancer and oral precancerous lesions and conditions—a clinical study*.* Indian journal of dentistry, **2**(2): p. 1-7; (2011).

35. Kumar, P., et al., Serum lipid profile in oral cancer and leukoplakia: correlation with tobacco abuse and histological grading*.* Journal of cancer research and therapeutics, **8**(3): p. 384-388; (2012).

36. Anuradha, A., et al., Lipid profile in oropharyngeal cancers in southern India*.* Journal of cancer research updates, **2**(2): p. 99-104; (2013).

37. Kumar, P., Singh, A., Sankhla, B. and Naraniya, A., Alteration in plasma lipid profile in oral submucous fibrosis patients: A case control study*.* South Asian journal of cancer, **2**(3): p. 147; (2013).

38. Singh, S., Ramesh, V., Premalatha, B., Prashad, K.V. and Ramadoss, K., Alterations in serum lipid profile patterns in oral cancer*.* Journal of natural science, biology, and medicine, **4**(2): p. 374; (2013).

39. Srinivas, G.V., Namala, S., Ananthaneni, A., Puneeth, H.K. and Devi, B.S., Evaluation and correlation of serum lipid profile in oral and gastrointestinal cancer patients*.* Journal of international oral health: JIOH, **5**(6): p. 72; (2013).

40. Ajai, K., et al., Estimation of serum lipids in patients with oral submucous fibrosis in India*.* Journal of clinical and experimental dentistry, **6**(3): p. e237; (2014).

41. Banseria, N., et al., Correlation of serum lipid profile, serum calcium, alkaline phosphatase and serum protein with histopathological grading and staging in head and neck cancer*.* J evol med dent sci, **3**: p. 1978-86; (2014).

42. Ganavi, B.S., Evaluation of serum lipids and lipoproteins as prognosticators in leukoplakia*.* The journal of contemporary dental practice, **15**: p. 294-299; (2014).

43. Garg, D., et al., Serum lipid profile in oral precancer and cancer: a diagnostic or prognostic marker? Journal of international oral health: JIOH, **6**(2): p. 33; (2014).

44. Goel, P., Garg, R. and Raghavan, V., Lipid profile in oral potentially malignant disorders*.* Journal of Indian academy of oral medicine and radiology, **26**(4): p. 374-378; (2014).

45. Kamath, A., Shashidhar, K.N., Anantharamaiah, H., Rangareddy, H. and Sathyanarayana, V.B., Risk factors, lipid profile, and histopathological study of oral cancers in Kolar district: a case-control study*.* Journal of cancer research and therapeutics, **10**(1): p. 171-175; (2014).

46. Mahesh, N., et al., Alterations of plasma lipid profile patterns in oral leukoplakia*.* Journal of international oral health: JIOH, **6**(1): p. 78; (2014).

47. Mehta, R., Gurudath, S., Dayansoor, S., Pai, A. and Ganapathy, K., Serum lipid profile in patients with oral cancer and oral precancerous conditions*.* Dental research journal, **11**(3): p. 345; (2014).

48. Neerupakam, M., et al., Alterations in plasma lipid profile patterns in oral cancer*.* Journal of Indian academy of oral medicine and radiology, **26**(3): p. 274-278; (2014).

49. Radhakrishna M.D.S, M., Alteration in serum lipid profile patterns in oral submucous fibrosis patients-A cross sectional study*.* Health sciences; (2014).

50. Sasankoti, N.G.D.R.P., Verma, M.D.S. and Sankar, N., Alterations in serum lipid profile patterns in head & neck cancer and oral submucous fibrosis patients*.* International dental journal of students research, **2**: p. 17-24; (2014).

51. Agarwal, A., Saxena, A., Rubens, M., Ahuja, R. and Tijare, M., Exploratory study to evaluate changes in serum lipid levels as early diagnostic and/or prognostic Indicators for oral submucous fibrosis and cancer among gutkha consumers in India*.* Asian pacific journal of cancer prevention, **16**(15): p. 6439-6444; (2015).

52. Dongre, C.A., Bagdey, S., Dive, A. and Dongre, U.J., Evaluation of lipid profile in oral submucous fibrosis and oral squamous cell carcinoma patients*.* Int J pharm sci rev res, **33**(1): p. 136-139; (2015).

53. Kanthem, R.K. and Guttikonda, V.R., Serum lipid profile in oral submucous fibrosis: A clinico pathological study*.* Journal of oral and maxillofacial pathology, **19**(2): p. 139-144; (2015).

54. Acharya, S., Rai, P., Hallikeri, K., Anehosur, V. and Kale, J., Serum lipid profile in oral squamous cell carcinoma: alterations and association with some clinicopathological parameters and tobacco use*.* International journal of oral and maxillofacial surgery, **45**(6): p. 713-720; (2016).

55. Li, G., et al., Alteration of serum lipid profile and its prognostic value in head and neck squamous cell carcinoma*.* Journal of oral pathology & medicine, **45**(3): p. 167-172; (2016).

56. Mujoo, S., Shashikant, M.C. and Dubey, A., Correlation of lipid profile and glycemia in oral precancerous lesion and conditions*.* Group, **37**(7.194): p. 21-48; (2016).

57. Poorey, V.K. and Thakur, P., Alteration of lipid profile in patients with head and neck malignancy*.* Indian journal of otolaryngology and head & neck surgery, **68**: p. 135-140; (2016).

58. Reddy, A.V., et al., Analysis of lipid profile in cancer patients, smokers, and nonsmokers*.* Dental research journal, **13**(6): p. 494-499; (2016).

59. Vyas, T., Bhargava, R. and Sharma, A., Comparative study of serum lipid profile parameters for oral cancer and non oral cancer patients*.* Int J com health and med res, **2**(2): p. 49-55; (2016).

60. Bhagat, A., et al., Lipid profile estimation in patients with oral premalignant disorders, and oral cancer-a comparative study*.* Journal of advanced medical and dental sciences research, **5**(4): p. 91; (2017).

61. Swamy, K.M. and Ganiger, A., Alterations of serum lipid profile in oral submucous fibrosis*.* Int J otorhinolaryngol head neck surg, **3**: p. 1064-7; (2017).

62. Anand, K., Sudheer, A. and Chatterjee, K., Alteration in serum lipid profile pattern in oral cancer and oral submucous fibrosis patients*.* Journal of Indian academy of oral medicine and radiology, **30**(1): p. 38-40; (2018).

63. Pratap, M., Sinha, A., Srivastav, S., Mishra, A. and Iqbal, H., Estimation of serum lipid profile in patients with OSMF*.* Journal of Indian academy of oral medicine and radiology, **30**(2): p. 102-106; (2018).

64. Yashveer, J. and Pankaj, D., Alteration of serum lipid in oral sub mucous fibrosis in central India*.* Indian journal of otolaryngology and head & neck surgery, **70**: p. 421-424; (2018).

65. Vijay, P., et al., Serum lipid profile analysis in patients with oral leukoplakia*.* Adv res J multidiscip discov, **33**: p. 21-24; (2019).

66. Vyas, T., Kuthiala, P., Sarathy, N., Bansal, A. and Singla, S., Evalution of serum lipid profile in head and neck cancer, OSMF and leukopkia patients*.* Journal of advanced medical and dental sciences research, **7**(8): p. 10-19; (2019).

67. Ramesh, S., Govindaraju, P. and Pachipalusu, B., Evaluation of serum lipid profile and complete hemogram in patients with oral submucous fibrosis: a case control study*.* Journal of international oral health, **12**(5): p. 427-431; (2020).

68. Agrawal, A.G., et al., Alteration in serum lipid profile pattern in oral squamous cell carcinoma and potentially malignant disorders*.* Pesquisa brasileira em odontopediatria e clínica integrada, **21**: p. e0083; (2021).

69. Siddique, S., et al., Evaluation of serum lipid profile in patients suffering from oral submucous fibrosis*.* Pakistan journal of medical & health sciences, **15**(10): p. 2793-2795; (2021).

70. Singh, V., et al., Diagnostic significance of serum and salivary lipid levels in oral precancer and oral cancer*.* National journal of maxillofacial surgery, **12**(2): p. 188-192; (2021).

71. Alazzawi, W., et al., The evaluation of serum lipid profile and apolipoprotein C-1 in the Iranian patients of oral squamous cell carcinoma*.* Biomedicine, **12**(3): p. 40; (2022).

72. Chowdhury, S., Kaushal, A., Mahendra, A. and Upadhayaya, N., Lipid profile estimation in oral malignant and premalignant lesions*.* International journal of scientific research: p. 36-38; <https://doi.org/10.36106/ijsr/3609104> (2022).

73. Khyalia, S., A comparative study of serum lipid profile in patients with oral squamous cell carcinoma and healthy population in a tertiary health care centre in north-western India*.* Asian pacific journal of cancer biology, **7**(3): p. 239-244; (2022).

74. Sachin, B., et al., "Serum lipid profile in patients with oral potentially malignant disorders*.* International journal of early childhood special education, **14**: p. 2022; <https://doi.org/10.9756/INT-JECSE/V14I4.68> (2022).

75. Sai, S.K., et al., Lipid profile in oral potentially malignant disorders and oral squamous cell carcinoma–A prognostic view*.* Journal of oral and maxillofacial pathology, **26**(4): p. 464-469; (2022).

76. Vikram, B. and Dharwadkar, A.A., Biochemical profile alterations of lipids in oral submucous fibrosis*.* International journal of advanced biochemistry research, **6**(2): p. 127-129; <https://doi.org/10.33545/26174693.2022.v6.i2b.149> (2022).

77. Adnan, S., Youssef, Y. and Mostafa, I., Alterations of serum lipids profiles in patients with head and neck cancers*.* International journal of cancer and clinical research, **10**; <https://doi.org/10.23937/2378-3419/1410181> (2023).

78. Rashmi, K., Naik, V., Kini, R. and Goyal, S., Serum lipid profile: A cellular signature in occurrence and progression of potentially malignant disorders and oral cancer*.* Journal of oral medicine, oral surgery, oral pathology and oral radiology, **4**(1): p. 14-20; (2023).

79. Qian, L., et al., Clinical relevance of serum lipids in the carcinogenesis of oral squamous cell carcinoma*.* BMC oral health, **23**(1): p. 200; (2023).

80. Varshney, S., Agarwal, P., Kumar, L., Tyagi, S. and Gupta, S., Study of serum lipid profile in patients of oral squamous cell carcinoma*.* Journal of Dr. YSR university of health sciences, **12**(3): p. 205-210; (2023).

81. Chandra, A., et al., Comparison of haematological and serum lipid profile parameters in oral potentially malignant disorders and oral squamous cell carcinoma*.* National journal of maxillofacial surgery, **16**(2): p. 263-270; (2025).
